# Enhanced Production of Photosynthetic Pigments and Various Metabolites and Lipids in the Cyanobacteria *Synechocystis* sp. PCC 7338 Culture in the Presence of Exogenous Glucose

**DOI:** 10.3390/biom11020214

**Published:** 2021-02-03

**Authors:** YuJin Noh, Hwanhui Lee, Myeongsun Kim, Seong-Joo Hong, Hookeun Lee, Dong-Myung Kim, Byung-Kwan Cho, Choul-Gyun Lee, Hyung-Kyoon Choi

**Affiliations:** 1 College of Pharmacy, Chung-Ang University, Seoul 06974, Korea; n5uuu@naver.com (Y.N.); hwanhui56@gmail.com (H.L.); myeongsunkim0242@gmail.com (M.K.); 2 Department of Biological Engineering, Inha University, Incheon 22212, Korea; owlet77@gmail.com (S.-J.H.); leecg@inha.ac.kr (C.-G.L.); 3 College of Pharmacy, Gachon University, Incheon 13120, Korea; hklee@gachon.ac.kr; 4 Department of Chemical Engineering and Applied Chemistry, Chungnam National University, Daejeon 34134, Korea; dmkim@cnu.ac.kr; 5 Department of Biological Sciences, Korea Advanced Institute of Science and Technology (KAIST), Daejeon 34141, Korea; bcho@kaist.ac.kr

**Keywords:** *Synechocystis* 7338, exogenous glucose, photosynthetic pigments, metabolic profiling, lipidomic profiling, GC-MS, nanoESI-MS

## Abstract

*Synechocystis* strains are cyanobacteria that can produce useful biomaterials for biofuel and pharmaceutical resources. In this study, the effects of exogenous glucose (5-mM) on cell growth, photosynthetic pigments, metabolites, and lipids in *Synechocystis* sp. PCC 7338 (referred to as *Synechocystis* 7338) were investigated. Exogenous glucose increased cell growth on days 9 and 18. The highest production (mg/L) of chlorophyll a (34.66), phycocyanin (84.94), allophycocyanin (34.28), and phycoerythrin (6.90) was observed on day 18 in *Synechocystis* 7338 culture under 5-mM glucose. Alterations in metabolic and lipidomic profiles under 5-mM glucose were investigated using gas chromatography-mass spectrometry (MS) and nanoelectrospray ionization-MS. The highest production (relative intensity/L) of aspartic acid, glutamic acid, glycerol-3-phosphate, linolenic acid, monogalactosyldiacylglycerol (MGDG) 16:0/18:1, MGDG 16:0/20:2, MGDG 18:1/18:2, neophytadiene, oleic acid, phosphatidylglycerol (PG) 16:0/16:0, and PG 16:0/17:2 was achieved on day 9. The highest production of pyroglutamic acid and sucrose was observed on day 18. We suggest that the addition of exogenous glucose to *Synechocystis* 7338 culture could be an efficient strategy for improving growth of cells and production of photosynthetic pigments, metabolites, and intact lipid species for industrial applications.

## 1. Introduction

Cyanobacteria, blue-green algae, have been used as an alternative biofuel resource with advantages over crops such as high growth rate, photosynthetic efficiency, and ability to utilize non-arable land and water from various sources [1]. Cyanobacteria grow photoautotrophically, converting solar energy into chemical and bioenergy via CO_2_ fixation. Cyanobacterial cultivation with exogenous organic carbon sources, such as glucose, sucrose, acetate, and glycerol, has been shown to be more advantageous for the production of biomass and biomaterials than that under autotrophic conditions [2,3]. Glucose is the most commonly used exogenous carbon source in photoautotrophic organisms, microalgae, and cyanobacteria to increase biomass and valuable compounds [4,5,6,7].

Cyanobacteria can produce resources used for food, feed, and pharmaceuticals [8]. *Synechocystis* sp. PCC 6803 (referred to as *Synechocystis* 6803) is the first cyanobacterium species whose entire genome sequence has been elucidated [9,10]. *Synechocystis* 6803 produces high-value biomaterials, such as phycobiliproteins, fatty acids, bioethanol, and alkanes [11,12]. *Synechocystis* 6803 that was engineered metabolically to synthesize isobutanol showed an increased isobutanol content after adding exogenous glucose, when compared with photoautotrophic conditions [13]. However, one of the *Synechocystis* strains, *Synechocystis* sp. PCC 7338 (referred to as *Synechocystis* 7338), has not been extensively studied. In our previous preliminary study, we observed that *Synechocystis* 7338 showed a lower growth rate and a higher production of useful biomaterials per cell than *Synechocystis* 6803 [14]. Although there is limited information on *Synechocystis* 7338, it is a species with the potential to produce several useful compounds and hence deserves further investigation.

Phycobiliproteins are colored proteins that play a role in light-harvesting and energy transfer to chlorophyll in the cyanobacteria photosynthetic apparatus [15]. These are broadly classified into three primary types: phycocyanin (PC), allophycocyanin (APC), and phycoerythrin (PE) [15,16]. Phycobiliproteins are widely studied as bioactive compounds with antioxidant, anticancer, anti-inflammatory, and antiviral activities that promote human health [15,17]. Furthermore, they are used as analytical reagents to develop fluorescent probes that can be adapted for immunohistochemistry, flow cytometry, and confocal laser microscopy [17]. There is a demand for increased phycobiliprotein production owing to their valuable properties in diverse research and industry fields.

Metabolic profiling of microalgae species has been performed using gas chromatography-mass spectrometry (GC-MS) [18,19], whereas lipidomic profiling has been conducted using nanoelectrospray ionization-MS (nanoESI-MS) in plants, microalgae, and cyanobacteria [20,21,22]. Both types of profiling have been used to understand cyanobacterial characteristics in altered environments, such as nutrient starvation and salinity stress [23,24]. Both organic carbon sources and light are supplied as energy sources under mixotrophic conditions, whereas only carbon sources are supplied under heterotrophic culture conditions. Integrated proteomic and lipidomic study using liquid chromatography-MS (LC-MS) in *Synechocystis* 6803 grown under light-activated heterotrophic conditions vs. mixotrophic conditions was conducted [25]. Transcriptomic and metabolomic analyses, focused on glycolysis and the tricarboxylic acid (TCA) cycle in *Synechocystis* 6803, have also been performed using capillary electrophoresis time-of-flight MS under various trophic conditions [26]. However, the effects of exogenous glucose on metabolic and lipidomic profiles in *Synechocystis* strains remain to be investigated.

Therefore, the present study investigated the effects of exogenous glucose on the growth and photosynthetic pigment content of *Synechocystis* 7338. *Synechocystis* 6803 did not grow well in the presence of 5-mM glucose in complete darkness or without 5-mM glucose under light-pulsed conditions (5 min of white light of 40 μmol m^−2^ s^−1^) in a previous study [27]. The glucose concentration of 5-mM is widely used to investigate alterations in gene, transcripts, proteins, and metabolites in *Synechocystis* 6803 [26,28]. Therefore, we employed 5-mM glucose to investigate alterations in cell growth and various metabolites/intact lipid species in *Synechocystis* 7338 culture. Concerning industrial applications, the early- and late-stationary growth phases during which sufficient amounts of *Synechocystis* 7338 cells were secured were set as monitoring times. Thus, we investigated alterations in cell growth, photosynthetic pigments, metabolites, and lipids of *Synechocystis* 7338 on the days of early- and late-stationary phases (days 9 and 18) under glucose conditions. In addition, GC-MS and nanoESI-MS were employed to investigate the effect of exogenous glucose on the metabolic and lipidomic profiles in the *Synechocystis* 7338 culture. This study could help pave the way for using exogenous glucose to enhance the growth of *Synechocystis* species and production of valuable compounds from them.

## 2. Materials and Methods

### 2.1. Culture Conditions and Sample Collection

*Synechocystis* 7338 was obtained from the Pasteur Culture Collection, Pasteur Institute (Paris, France). The cells were cultivated in a modified artificial seawater nutrient (ASN III) medium that contained 25 g/L NaCl, 3.5 g/L MgSO_4_·7H_2_O, 2 g/L MgCl_2_·6H_2_O, 0.5 g/L KCl, 0.5 g/L CaCl_2_·2H_2_O, 0.75 g/L NaNO_3_, 20 mg/L K_2_HPO_4_·3H_2_O, 3 mg/L citric acid, 5 mg/L ethylenediaminetetraacetic acid (EDTA)·Na·2H_2_O, 2 mL/L trace metal mix A5 with cobalt (Sigma-Aldrich, St. Louis, MO, USA), 3 mg/L ammonium iron citrate, and 40 mg/L Na_2_CO_3_. The cells were cultured in 250 mL Erlenmeyer flasks that contained 100 mL of ASN III medium. The flasks were incubated in a shaking incubator (NEX220SRL, Nexus Technologies, Seoul, Republic of Korea) at 30 ± 1°C and 120 rpm under 40 *μ*mol photons m^−2^ s^−1^ and 16-h light/8-h dark cycles. To investigate the effect of exogenous glucose, 5-mM glucose was added to each flask. The experiment was performed in biological triplicates for the control and glucose-treated samples. Cells were collected on days 9 and 18 and washed thrice with 0.5-M ammonium formate solution (Sigma-Aldrich) via centrifugation and resuspension [29]. The collected cells were dried using a freeze-dryer (IlShinBioBase, Dongducheon city, Kyunggi-do, Republic of Korea) and stored at -80°C until further analysis.

### 2.2. Measurements of Cell Growth 

Cell growth was determined by measuring the optical density (OD) of the cell cultures at 730 nm using a microplate spectrophotometer (Bio-Rad, Hercules, CA, USA). Cell growth was measured daily in triplicate.

### 2.3. Measurement of Chlorophyll a Content

Chlorophyll a content in *Synechocystis* 7338 culture was determined on days 9 and 18, using a previously described method [30]. In brief, 1 mL cell culture from each sample was centrifuged at 10,000× *g* and 4 °C for 10 min, and 0.9 mL supernatant was removed. Subsequently, 0.9 mL of methanol was added to the cell pellets, followed by refrigeration for 1 h in the dark. The OD of the methanolic extract was measured at 665 nm using a microplate spectrophotometer. The OD_665_ was converted into chlorophyll a content using the following equation:

(1) $$\mathrm{Chlorophyll}\;a\;(\mathrm{mg}/L)={\mathrm{OD}}_{665}\times 13.9$$

### 2.4. Measurement of Phycobiliprotein Content

Phycobiliprotein content in *Synechocystis* 7338 culture was determined on days 9 and 18 using a previously described method with some modifications [31]. Briefly, 1 mL cell culture from each sample was centrifuged at 10,000× *g* and 4 °C for 10 min. Then, 0.9 mL of the supernatant was removed. The residue was homogenized using an ultrasonic cell homogenizer (HD2070, Bandelin, Germany) for 2 min in an ice bath. Thereafter, 0.9 mL of 20 mM sodium acetate buffer at pH 5.5 and 0.2 mL of streptomycin sulfate were added to the residue and briefly vortexed. To facilitate precipitation, resuspended cells were incubated at 4 °C for 30 min and centrifuged at 10,000× *g* and 4 °C for 10 min. The OD of the extract was measured at 620, 650, and 565 nm using the microplate spectrophotometer. The PC, APC, and PE contents were calculated using the following equations:

(2) $$\mathrm{PC}\;\left(\mathrm{mg}/L\right)=(\frac{{\mathrm{OD}}_{620}-{0.7\times \mathrm{OD}}_{650}}{7.38})\times 10^{3}$$

(3) $$\mathrm{APC}\;\left(\mathrm{mg}/L\right)=(\frac{{\mathrm{OD}}_{650}-{0.19\times \mathrm{OD}}_{620}}{5.65})\times 10^{3}$$

(4) $$\mathrm{PE}\;\left(\mathrm{mg}/L\right)=(\frac{{\mathrm{OD}}_{565}-2.8\;\left(\mathrm{PC}\right)\;-1.34\;(\mathrm{APC})}{12.7})\times 10^{3}$$

### 2.5. Measurement of the Maximum Quantum Yield (QYmax) of Photosystem II

QY_max_ of photosystem II, which was measured to characterize the photosynthetic activity, was determined from the chlorophyll fluorescence of dark-adapted cells using an AquaPen-C AP-C 110 fluorometer (Photon Systems Instruments, Drasov, Czech Republic) according to a previously described method [32].

### 2.6. Metabolite Analysis Using GC-MS 

Five milligrams of each *Synechocystis* 7338 sample from days 9 and 18 were weighed and used for metabolic profiling by GC-MS, according to a previously described method [33]. A gas chromatography system (7890A, Agilent Technologies, CA, USA) equipped with a mass selective detector (5975C, Agilent Technologies) and an autosampler (7683B, Agilent Technologies) was used in the present study. Myristic acid -*d*_27_ (Sigma-Aldrich) was used as an internal standard (IS) at 1000 mg/L. GC-MS analysis was performed in triplicate. To ensure the quality of the analytical method using GC-MS, 10 μL of the extract from each sample was collected for quality control (QC) and analyzed in nonuplicate.

### 2.7. Lipid Analysis Using nanoESI-MS

Two milligrams of each *Synechocystis* 7338 sample from days 9 and 18 were weighed and used for lipidomic profiling by nanoESI-MS, following a previously described method [34]. A linear ion-trap mass spectrometer (LTQ-XL, ThermoFisher Scientific, San Jose, CA, USA) coupled with an automated nanoinfusion/nanospray source (Triversa NanoMate System, Advion Biosciences, Ithaca, NY, USA) was used. NanoESI-MS analysis was performed in triplicate. To ensure the quality of the analytical method using nanoESI-MS, 10 μL of the extract from each sample was collected for QC and analyzed in nonuplicate. 

### 2.8. Data Processing and Multivariate Analysis

To process the raw data from GC-MS and nanoESI-MS analyses and identify metabolites and intact lipid species, we used previously described methods [33,34]. The Student’s *t*-test and Mann–Whitney test were used to evaluate significant differences in relative level (relative intensity/g) obtained from GC-MS and nanoESI-MS. The significance level was set at *p* < 0.05, and the analysis was run using SPSS statistics software (ver. 25, IBM, Somers, NY, USA). The SIMCA software (ver. 15.0.2, Sartorius Stedim Data Analytics AB, Umea, Sweden) was used to ensure the validity of the instrument methods, and Pareto scaling was applied to construct principal component analysis (PCA) and partial least squares-discriminant analysis (PLS-DA)-derived score plots. The relative yields (relative intensity/L) were calculated using the following equation:

(5) $$\mathrm{Relative}\;\mathrm{yield}\;\left(\mathrm{relative}\;\mathrm{intensity}/L\right)=\left(C\times \mathrm{DCW}\right)$$

where C is the relative level of metabolites and intact lipid species (relative intensity/g), and DCW is the dry cell weight (g/L).

## 3. Results

### 3.1. Effect of Exogenous Glucose on the Growth of Synechocystis 7338

**Figure 1:**
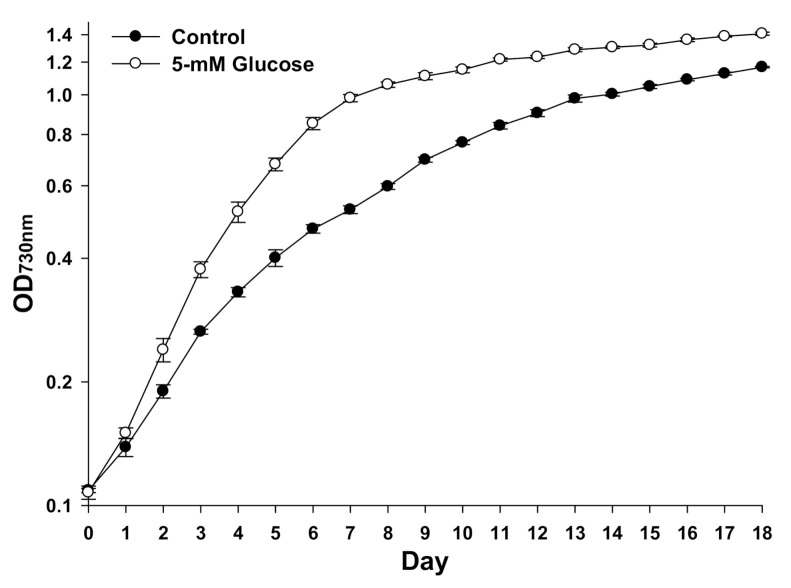
Growth of *Synechocystis* 7338 under control and 5-mM exogenous glucose conditions (*n* = 9, biological triplicates and experimental triplicates). Data represents the means of biological and experimental triplicates, and error bars indicate the standard deviations.

As shown in Figure 1, *Synechocystis* 7338 cultured with 5-mM glucose showed a higher growth than the control. Biomass production (dry weight, mg/L) was significantly higher on day 9 (99.5% increase) and 18 (53.8% increase) following exogenous glucose treatment than the control (data not shown).

### 3.2. Effect of Exogenous Glucose on Chlorophyll a and Phycobiliprotein Content of Synechocystis 7338

**Figure 2:**
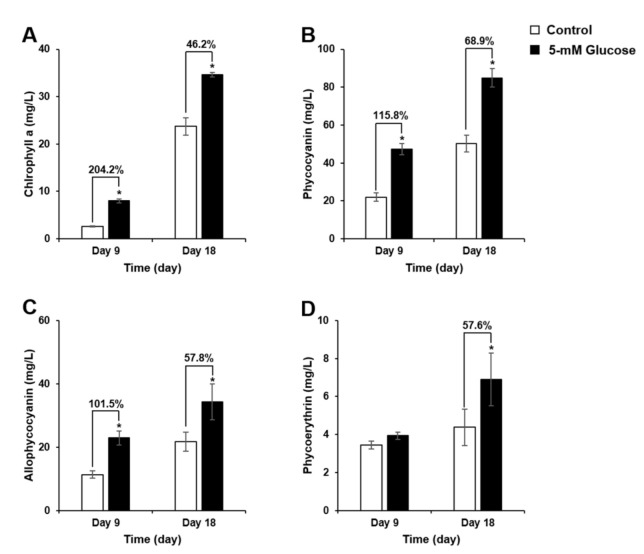
Pigment content of *Synechocystis* 7338 under control and 5-mM exogenous glucose treatments (*n* = 9, biological and experimental triplicates). Chlorophyll a (**A**), phycocyanin (**B**), allophycocyanin (**C**), and phycoerythrin (**D**). Data represent the means of biological and experimental triplicates, and error bars indicate the standard deviation. The asterisk (*) indicates a significant difference between the control and 5-mM glucose groups as determined by the Mann–Whitney test (*p* < 0.05). Percent changes indicate the increase in pigment content in the 5-mM glucose group compared with that in the control group, when there was a significant difference.

During *Synechocystis* 7338 culture, exogenous glucose significantly increased chlorophyll a content to 8.00 mg/L (204.2% increase) and 34.66 mg/L (40.2% increase) on days 9 and 18, respectively, compared with the control (Figure 2A).

PC and APC contents were significantly enhanced on days 9 and 18, whereas PE content significantly increased on day 18. As shown in Figure 2B,C, the highest rates of increase in PC (115.8%) and APC (101.5%) were observed on day 9. In addition, exogenous glucose treatment resulted in the highest PC (84.94 mg/L), APC (34.28 mg/L), and PE (6.90 mg/L) levels on day 18 (68.9, 57.8, and 57.6% increase, respectively) in *Synechocystis* 7338 culture (Figure 2B–D).

### 3.3. Effect of Exogenous Glucose on Photosynthetic Activity of Synechocystis 7338

As shown in Table 1, the QY_max_ values were measured on days 1, 9, and 18 after addition of 5-mM glucose and compared with those of the control group. QY_max_ was calculated using the ratio of the variable to maximum fluorescence (Fv/Fm), which is considered a sensitive indicator of photosynthetic activity [35]. The cells exposed to glucose early in the trial (day 1) showed a slight disturbance in photosynthetic activity (QY_max_, 90%). However, long-term (days 9 and 18) exposure of *Synechocystis* 7338 to glucose showed full recovery of photosynthetic activity, which was subsequently maintained (QY_max_, 100% and 102%, respectively).

### 3.4. Effect of Exogenous Glucose on Comprehensive Metabolic and Lipidomic Profiles of Synechocystis 7338

**Figure 3:**
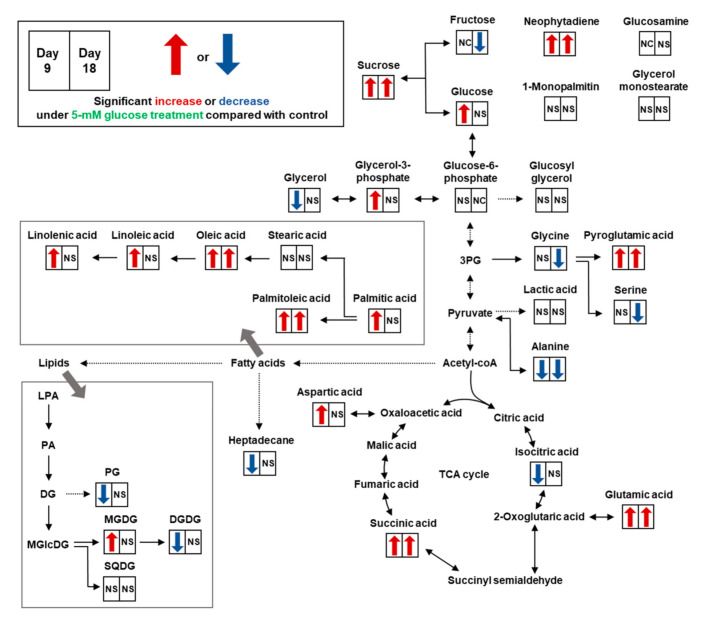
Comprehensive pathway representing the change in relative levels of metabolites and intact lipid species in *Synechocystis* 7338 in response to 5-mM exogenous glucose treatment. The diagram is based on the pathway in the KEGG database (http://www.kegg.jp/kegg/). The total abundance of lipid species in each class was used. NS, non-significant compared with the control; NC; non-calculable; 3PG, 3-phosphoglyceric acid; LPA, lysophosphatidic acid; PA, phosphatidic acid; DA, diacylglycerol; PG, phosphatidylglycerol; MGlcDG, monoglucosylglycerol; MGDG, monogalactosyldiacylglycerol; DGDG, digalactosyldiacylglycerol; SQDG, sulfoquinovosyldiacylglycerol.

As shown in Table S1, 27 metabolites were identified as follows: two alcohols, six amino acids, six fatty acids, two glycerolipids, three organic acids, six sugars, and two others. Significantly different levels of 17 metabolites on day 9 and 11 metabolites on day 18 were observed in the 5-mM glucose and control groups (Table 2). On day 9, the relative levels of most fatty acids were significantly higher in the glucose-treated group than in the control group. On day 18, however, only two fatty acids significantly increased in response to glucose addition. Compared with the control, 5-mM glucose significantly increased the levels of glutamic acid, pyroglutamic acid, succinic acid, sucrose, and neophytadiene on days 9 and 18 (Figure 3).

Table 3 presents the relative levels of various intact lipid species (per cell) in *Synechocystis* 7338. A total of 50 intact lipid species were identified, which were as follows: 6 digalactosyldiacyglycerols (DGDGs), 13 monogalactosyldiacylglycerols (MGDGs), 2 phytyl derivatives, 7 phosphatidylglycerols (PGs), and 22 sulfoquinovosyldiacylglycerols (SQDGs). The representative MS/MS spectra of nine intact lipid species with an odd number of fatty acyl chains (MGDG 16:0/17:2, MGDG 16:0/21:0, PG 16:0/17:2, PG 16:0/17:1, SQDG 16:0/17:2, SQDG 16:0/17:1, SQDG 16:0/17:0, SQDG 17:0/18:2 and SQDG 16:0/19:0) were shown in Figure S1A–I.

Significantly different levels of 37 intact lipid species on day 9, and 15 intact lipid species on day 18, were observed in the 5-mM glucose and control treatment groups. Although a number of intact lipid species showed a decreasing trend under the 5-mM glucose treatment, the relative levels of DGDG 18:2/18:3, six MGDGs (16:0/18:1, 18:2/18:3, 18:2/18:2, 18:1/18:2, 16:0/20:2, and 16:0/20:1), two PGs (16:0/16:0 and 16:0/17:2), and three SQDGs (16:0/18:1, 16:0/18:0, and 18:0/18:1) significantly increased in the 5-mM glucose treatment group on day 9. On day 18, the relative levels of DGDG 16:0/18:1, MGDG 16:0/18:1, two phytyl derivatives (pheophytin a and chlorophyll a), two PGs (16:0/16:0 and 16:0/18:0), and SQDG 16:0/20:0 significantly increased in the 5-mM glucose treatment group.

As shown in Figure 3, the total levels of lipid species in each lipid class showed a significant change on day 9 in *Synechocystis* 7338 exposed to 5-mM glucose. The total relative levels of DGDG and PG significantly decreased in *Synechocystis* 7338 under exogenous glucose treatment compared with the levels of those under the control. In contrast, MGDG exhibited a significant increase in glucose-treated *Synechocystis* 7338. On day 18, there was no difference in total lipid abundance between the 5-mM glucose and control treatments.

### 3.5. Multivariate Statistical Analysis Based on GC-MS and nanoESI-MS Analyses of Synechocystis 7338 Treated with Exogenous Glucose

In the PCA-derived score plots (Figure S2), clustered QC samples suggest that GC-MS and nanoESI-MS analyses were technically valid. PLS-DA-derived score plots were constructed using the profiles of metabolites and intact lipid species of *Synechocystis* 7338 (Figure S3A,B). Cross-validation (R^2^Y and Q^2^Y) was performed to ensure model fitness and predictivity of the parameters to establish a better model (Figure S3C,D). These values are better indicators when the value is closer to 1 [36]. In Figure S3A,B, the PLS-DA-derived score plot showed clear separation with valid cross-validation values between the 5-mM glucose and control groups on day 9 (R^2^Y, 0.971; Q^2^Y, 0.974) and 18 (R^2^Y, 0.928; Q^2^Y, 0.947). In addition, a permutation test (R^2^Y intercept and Q^2^Y intercept) was performed to validate the PLS-DA models. Values below 0.3–0.4 and 0.05 were considered valid for the R^2^Y intercept and Q^2^Y intercept, respectively. PLS-DA models between the 5-mM glucose and control groups on days 9 and 18 had acceptable values of R^2^Y intercept, 0.326, Q^2^Y intercept, −0.548; and R^2^Y intercept, 0.337, Q^2^Y intercept, −0.354 on respective days (Figure S3C,D).

**Figure 4:**
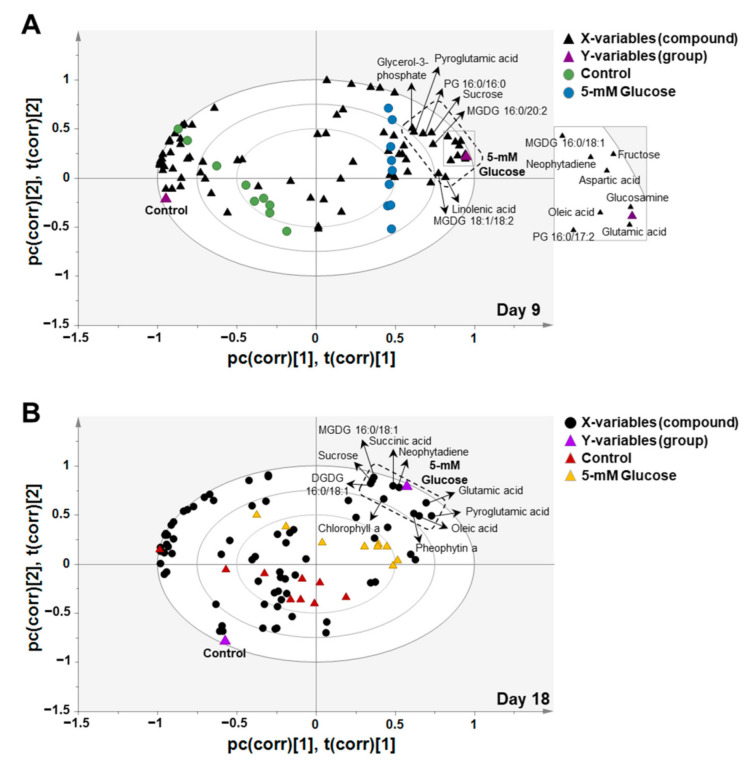
Biplots derived from partial least squares-discriminant analysis (PLS-DA) with samples from days 9 (**A**) and 18 (**B**) of the control and 5-mM exogenous glucose treatments (*n* = 9, biological triplicates and experimental triplicates). PLS-DA-biplots indicate the correlation of compounds identified in the 5-mM exogenous glucose group (X-variables) and the group information (Y-variables).

As shown in Figure 4, the PLS-DA-biplots indicate correlation between X-variables (compound) and Y-variables (group) of days 9 and 18 samples. The compounds that have high explained variances were positioned to the outer ellipses, and the three ellipse lines provide explained variances of 50%, 75%, or 100% [37,38]. Compounds positioned closer to the outer ellipse on the PLS-DA-biplot are considered to make a more significant contribution to group separation. In addition, the closely located compounds and groups in Figure 4 represented a high correlation between them, whereas the more separated compounds and groups represented a low correlation. In this study, the PLS-DA-biplot showed that aspartic acid, fructose, glucosamine, glutamic acid, glycerol-3-phosphate, linolenic acid, MGDG 16:0/18:1, MGDG 16:0/20:2, MGDG 18:1/18:2, neophytadiene, oleic acid, PG 16:0/16:0, PG 16:0/17:2, pyroglutamic acid, and sucrose were positioned closer to the 5-mM glucose group on day 9 (Figure 4A). Chlorophyll a, DGDG 16:0/18:1, glutamic acid, MGDG 16:0/18:1, neophytadiene, oleic acid, pheophytin a, pyroglutamic acid, succinic acid, and sucrose were positioned closer to the 5-mM glucose group on day 18 (Figure 4B). These compounds noticeably discriminated between the 5-mM glucose and control groups on days 9 and 18.

### 3.6. Relative Yields (Relative Intensity/L) of Various Metabolites and Intact Lipid Species in Synechocystis 7338 Treated with Exogenous Glucose

As described previously, exogenous glucose addition increased the relative levels of various *Synechocystis* 7338 compounds. The selected compounds from the PLS-DA-biplot are represented as relative yields in Table 4. On day 9, glucose addition significantly enhanced the relative yields of aspartic acid (4.53-fold), glutamic acid (5.07-fold), glycerol-3-phosphate (2.89-fold), linolenic acid (3.74-fold), MGDG 16:0/18:1 (22.70-fold), MGDG 16:0/20:2 (2.67-fold), MGDG 18:1/18:2 (2.51-fold), neophytadiene (4.19-fold), oleic acid (9.23-fold), PG 16:0/16:0 (2.54-fold), PG 16:0/17:2 (3.65-fold), pyroglutamic acid (2.39-fold), and sucrose (3.80-fold). Fructose and glucosamine could not be compared because they were not identified in the control samples. Relative yields of MGDG 16:0/18:1 (3.40-fold), neophytadiene (1.33-fold), and sucrose (1.10-fold) were significantly increased in the 5-mM glucose treatment on day 18 (Table 4).

In addition, equally identified compounds with enhanced relative abundance in the 5-mM glucose group from the PLS-DA-biplots were compared between days 9 and 18 (Table 5). Relative yields of glutamic acid, MGDG 16:0/18:1, neophytadiene, and oleic acid were significantly higher on day 9, whereas pyroglutamic acid and sucrose levels were significantly higher on day 18. Further studies with higher temporal resolution are required to select the optimal harvest time for each compound in *Synechocystis* 7338 culture under 5-mM glucose treatment conditions.

## 4. Discussion

Cyanobacteria can grow under photoautotrophic, heterotrophic, and mixotrophic conditions and can utilize glucose as an exogenous organic carbon source [2]. Glucose is a widely used and relatively inexpensive material for microalgae and microbial culture to enhance biomass and synthesis of valuable compounds [39,40,41,42]. Chlorophyta have the ability to fix CO_2_ with higher efficiency than terrestrial plants via photosynthesis; microalgae can trap CO_2_ produced by heterotrophic growth that is then utilized for photoautotrophic growth [43]. The enhanced growth of *Synechocystis* 7338 in our study could be explained by the combined effects of autotrophic growth by photosynthesis and heterotrophic growth by the use of exogenous glucose and recycled CO_2_.

There have been contradictory results regarding the effect of exogenous glucose addition on chlorophyll a content. In *Spirulina platensis* and *Anabaena* spp., exogenous glucose addition increased chlorophyll a content [44,45]. However, the biosynthesis of chlorophyll a was reduced by exogenous glucose in microalgal consortia culture collected from wastewater around Hyderabad city in India [28]. The chlorophyll a content was decreased in diatom *Fragilaria* cells 24 h after glucose addition, which was suggested as a potential adaptive response of algae to minimize expenditure of biosynthetic energy and material [46]. Further, the effect of glucose addition on chlorophyll a content might vary according to the experimental setup and the organism being tested [46]. In our study, exogenous glucose addition maintained, rather than inhibited, photosynthesis of *Synechocystis* 7338 by increasing chlorophyll a content.

Phycobiliproteins are light-harvesting pigments consisting of PC, APC, and PE [15,16]. Through phycobilisome rods (located in PE and PC) and the core (located in APC), photosynthetic energy is transferred to chlorophyll during the photosynthetic reaction [15,16,47]. *Nostoc* sp. culture supplied with exogenous glucose (0.5–2.5 g/L) showed increased PC and PE production [48]. Sugarcane was the most effective carbon source of *Nostoc* sp. culture, increasing the phycobiliprotein production 12.5 times compared to that without a carbon source, whereas 3.0-fold and 4.5-fold increases in phycobiliprotein production were achieved under glucose and sucrose conditions, respectively [48]. Furthermore, the degradation rate of PC was retarded by adding glucose (2%, *w*/*v*) to a *Synechocystis* 6803 culture [49]. In the present study, the highest production of PC, APC, and PE was achieved on day 18 under 5-mM glucose treatment conditions. It implies that carbon source could be optimized to further increase the productivity of phycobiliproteins in *Synechocystis* 7338 culture in future. 

In the current study, the photosynthetic activity of *Synechocystis* 7338 significantly decreased during early exposure (day 1) to glucose. Reduced expression of the photosynthetic gene *rubisco* has been reported for *Galdieria sulphuraria* under glucose conditions, resulting in decreased photosystem II photochemical efficiency [50]. In *C. vulgaris* strain UAM 101, the apparent affinity of CO_2_ was reduced during CO_2_ fixation in the presence of glucose [51]. Moreover, glucose is known to induce osmotic stress in *Synechocystis* 6803 [52]; stress conditions might cause a reduction in photosynthetic efficiency of algae cells. Reduction of photosynthetic activity during early exposure to glucose might be caused by limited photosynthetic gene expression, reduced CO_2_ affinity_,_ or increased osmotic stress. However, in the current study, the photosynthetic activity fully recovered and was maintained at adequate levels after day 9 when cell growth stabilized. Previous report revealed that *Synechocystis* 7338 cells recovered their photosynthetic activity during long-term exposure to osmotic stress caused by increasing salinity [53]. Thus, *Synechocystis* 7338 strain might have the ability to endure osmotic stress induced by glucose addition, leading to recovery and maintenance of photosynthetic activity by enhancing photosynthetic pigments. 

Neophytadiene is a diterpenoid compound that possesses antimicrobial, anti-inflammatory, analgesic, antipyretic, and antioxidant activities [54,55,56]. Despite its versatility, previous studies have focused solely on extracting neophytadiene from several plants, and there are still no reports on how its content could be improved. Identification of neophytadiene in *Synechocystis* sp. and optimal methods for its extraction have been suggested [57]. Chromatographic isolation of neophytadiene with 99% purity was achieved by dichloromethane extraction followed by silica-gel column operation in tobacco sample [58]. From marine algae, *Turbinaria ornate*, neophytadiene was isolated using methanol extraction and silica-gel column chromatography [59]. To compare economic feasibility of various resources of neophytadiene, optimal isolation method should be estabilished in *Synechocystis* 7338 culture in future. The enhanced neophytadiene production in *Synechocystis* 7338 following exogenous glucose addition in the present study implied the potential applications in the pharmaceutical and nutraceutical industries.

Succinic acid has wide-ranging applications as an intermediate chemical in the food industry (perfume esters and flavors) and the chemical industry (surfactants and dyes) [60]. Increased metabolic flux of glycolysis and the TCA cycle has been observed in *Synechococcus* sp. PCC 7942 cultivation after the addition of glucose and acetate [61]. *Synechocystis* 6803 showed an increase in succinic acid level owing to TCA cycle activation by exogenous glucose [26]. Succinic acid is utilized in oxidative phosphorylation to produce ATP [62,63]. We deduced that increased succinic acid levels from the activated TCA cycle by exogenously supplied 5-mM glucose might contribute to enhanced ATP production through oxidative phosphorylation and subsequently enhance cell growth of *Synechocystis* 7338.

In previous reports, *Synechocystis* 6803 and *Neochloris oleoabundans* increased oleic acid content under nitrogen starvation conditions with relatively high carbon content in the medium [64,65]. Expression of the genes involved in oleic acid and linolenic acid synthesis was increased in *Botryococcus braunii* and *Messastrum gracile* grown under nitrogen-limited rather than nitrogen-repleted conditions [66,67]. We theorized that exogenously supplied glucose alters C:N ratio in the medium of *Synechocystis* 7338 culture, thereby enhancing the relative levels and yields of oleic acid and linolenic acid.

Under various environmental conditions of cyanobacteria growth, the lipid composition of the cell membrane changed [68]. The thylakoid lipids in cyanobacterial PS II include DGDGs, MGDGs, PGs, and SQDGs [68,69]. In addition, these lipid species from cyanobacteria possess anti-inflammatory and anti-thrombotic activities [70,71,72]. On day 9, a significant increase in the relative levels of intact lipid species was observed upon addition of exogenous glucose in our study. Cyanobacteria *Spirulina platensis* and *Nostoc linckia* were investigated for changes in lipid content against organic carbon sources such as 5- and 50-mM glucose and 5-mM mannose [73]. In the presence of glucose, an increase in PG content was observed in *S. platensis* and *N. linckia*. An increase in SQDG content was only observed in *N. linckia* when treated with 5-mM glucose. The lipid composition of cyanobacteria might be differently redistributed across species and culture periods in the presence of glucose. In addition, the increased levels of lipid species in PS II in long-term exposure to glucose might help to recover and maintain the photosynthetic activity which was inhibited in short-term exposure to glucose.

The relative level of palmitic acid (C16:0) was significantly increased on day 9 with 5-mM glucose compared with the control. Zili et al. [73] reported a change in the fatty acid profile of *Graesiella* sp. in mixotrophic (light + glucose) and heterotrophic (glucose) conditions, with C16:0 and saturated fatty acids being enhanced in the presence of glucose under both mixotrophic and heterotrophic conditions. C16:0 was considered an energy store in *Thalassiosira pseudonana*, *Phaeodactylum tricornutum*, and *Dunaliella tertiolecta* under conditions of excess energy; in this case, C16:0 would be oxidized to generate energy when needed [74,75]. In the present study, abundant C16:0 might have been used to synthesize DGDG, MGDG, and PG in *Synechocystis* 7338 in the presence of glucose. Further in vitro and/or in vivo studies are required for investigating the various bioactivities, including anti-inflammatory or anti-thrombotic activities, of the several intact lipid species enhanced in our study.

Future studies employing genomics, transcriptomics, and proteomics are needed to further investigate the molecular mechanism of exogenous glucose in increasing these metabolites and intact lipid species in *Synechocystis* 7338 culture.

## 5. Conclusions

This is the first study to investigate the effects of exogenous glucose on cell growth, photosynthetic pigments, and metabolic and lipidomic profiles of *Synechocystis* 7338. Distinct metabolic and lipidomic profiles of *Synechocystis* 7338 were determined using GC-MS and nanoESI-MS after adding exogenous glucose to the culture medium which resulted in enhanced cell growth, photosynthetic pigments, various metabolites, and intact lipid species in the *Synechocystis* 7338 culture. The addition of glucose to *Synechocystis* 7338 culture could be an easily applied and cost-effective strategy for enhancing the production of these materials for future industrial applications.

## Figures and Tables

**Table 1:** Changes in maximum quantum yield of photosystem II (QY_max_) of *Synechocystis* 7338 under the 5-mM exogenous glucose treatment conditions.

	Day 1	Day 9	Day 18
Control	0.50 ± 0.01	0.49 ± 0.01	0.48 ± 0.01
5-mM Glucose	0.45 ± 0.01 *	0.49 ± 0.01	0.49 ± 0.01
% Control	90	100	102

The asterisk (*) indicates significant differences (*p* < 0.05, from Student’s *t*-test) compared with control on day 1.

**Table 2:** Relative levels (relative intensity/g) ^1^ of metabolites identified in unit cells of *Synechocystis* 7338 under control and 5-mM exogenous glucose treatments determined by gas chromatography-mass spectrometry (GC-MS) analysis over two periods.

No.	Compound	Day 9		Day 18	
		Control	5-mM Glucose	Control	5-mM Glucose
	Alcohols				
1	Glycerol	2.10 ± 0.15	0.95 ± 0.17 *^,2^	1.76 ± 1.09	3.06 ± 3.25
2	Glycerol-3-phosphate	26.80 ± 6.47	39.07 ± 5.84 *	2.52 ± 2.92	6.65 ± 5.99
	Amino acids				
3	Alanine	23.18 ± 6.21	14.43 ± 1.11 *	11.07 ± 2.96	5.56 ± 2.75 *
4	Aspartic acid	27.31 ± 5.18	62.70 ± 10.30 *	48.06 ± 24.30	27.88 ± 18.00
5	Glutamic acid	44.90 ± 14.70	115.06 ± 8.47 *	52.21 ± 3.92	94.01 ± 14.52 *
6	Glycine	2.13 ± 0.49	1.74 ± 0.40	5.02 ± 0.37	3.54 ± 0.32 *
7	Pyroglutamic acid	5.79 ± 0.67	6.99 ± 0.98 *	7.19 ± 1.00	10.76 ± 1.49 *
8	Serine	0.39 ± 0.12	0.31 ± 0.03	0.87 ± 0.31	0.51 ± 0.22 *
	Fatty acids				
9	Linoleic acid	2.68 ± 0.78	3.46 ± 0.52 *	4.25 ± 0.66	4.96 ± 1.17
10	Linolenic acid	0.84 ± 0.23	1.58 ± 0.23 *	1.84 ± 0.42	1.83 ± 0.34
11	Oleic acid	2.02 ± 0.41	9.45 ± 1.94 *	5.01 ± 0.86	8.32 ± 1.89 *
12	Palmitic acid	17.99 ± 4.15	25.03 ± 4.83 *	39.84 ± 8.88	50.63 ± 11.27
13	Palmitoleic acid	0.31 ± 0.09	0.42 ± 0.09 *	0.68 ± 0.11	0.86 ± 0.20 *
14	Stearic acid	1.86 ± 0.31	2.06 ± 0.23	2.48 ± 0.39	2.49 ± 0.47
	Glycerolipids				
15	1-Monopalmitin	4.59 ± 0.92	6.09 ± 2.48	6.88 ± 2.78	7.32 ± 0.66
16	Glycerol monostearate	2.20 ± 0.40	2.94 ± 1.04	3.35 ± 1.33	3.41 ± 0.36
	Organic acids				
17	Isocitric acid	0.79 ± 0.08	0.54 ± 0.14 *	0.42 ± 0.24	0.64 ± 0.25
18	Lactic acid	0.98 ± 0.25	0.85 ± 0.26	1.01 ± 0.40	0.82 ± 0.14
19	Succinic acid	0.34 ± 0.11	0.79 ± 0.36 *	0.48 ± 0.10	2.29 ± 0.52 *
	Sugars				
20	Fructose	ND ^3^	0.43 ± 0.08	11.06 ± 0.60	6.43 ± 0.87 *
21	Glucosamine	ND	0.42 ± 0.02	3.71 ± 0.97	3.28 ± 0.56
22	Glucose	0.29 ± 0.06	0.36 ± 0.03 *	9.47 ± 5.72	20.23 ± 20.6
23	Glucose-6-phosphate	1.79 ± 1.18	0.34 ± 0.08	ND	ND
24	Glucosylglycerol	928.05 ± 102.33	947.57 ± 90.16	966.05 ± 97.71	960.70 ± 58.37
25	Sucrose	61.15 ± 26.60	117.98 ± 23.74 *	175.79 ± 22.35	296.63 ± 38.42 *
	Others				
26	Neophytadiene	5.20 ± 0.83	10.99 ± 1.11 *	4.45 ± 0.39	9.15 ± 0.96 *
27	Heptadecane	19.50 ± 3.84	13.41 ± 1.40 *	25.87 ± 6.68	22.97 ± 3.10

^1^ Values of metabolites are represented as means ± standard deviations (*n* = 9, biological triplicates and experimental triplicates) after normalization with an internal standard. ^2^ Asterisks (*) represent significant differences compared with each control on days 9 and 18, *p* < 0.05 (from Mann–Whitney test). ^3^ ND, not detected.

**Table 3:** Relative levels (relative intensity/g) ^1^ of intact lipid species identified in unit cells of *Synechocystis* 7338 under control and 5-mM exogenous glucose treatments determined by nanoESI-MS analysis over two periods.

No.	m/z	Lipid Species	Ion Species	Day 9		Day 18	
				Control	5-mM Glucose	Control	5-mM Glucose
	Positive ion mode						
	Digalactosyldiacylglycerol (DGDG)						
1	935	DGDG 16:1/18:3	[M+Na]^+^	33.75 ± 3.61	17.62 ± 4.36 *^,2^	30.17 ± 5.92	26.57 ± 2.90
2	937	DGDG 16:0/18:3	[M+Na]^+^	60.80 ± 14.09	35.98 ± 8.14 *	62.28 ± 8.63	65.06 ± 13.61
3	939	DGDG 16:0/18:2	[M+Na]^+^	41.13 ± 5.95	17.62 ± 5.01 *	39.36 ± 6.65	42.75 ± 10.33
4	941	DGDG 16:0/18:1	[M+Na]^+^	10.53 ± 2.72	10.01 ± 3.49	14.14 ± 2.38	22.72 ± 3.01 *
5	943	DGDG 16:0/18:0	[M+Na]^+^	53.69 ± 37.46	56.22 ± 16.97	71.58 ± 22.68	57.52 ± 7.93
6	961	DGDG 18:2/18:3	[M+Na]^+^	9.48 ± 1.76	12.15 ± 1.89 *	11.34 ± 3.27	10.09 ± 1.90
	Monogalactosyldiacylglycerol (MGDG)						
7	747	MGDG 14:0/18:3	[M+Na]^+^	29.11 ± 6.61	30.27 ± 2.04	32.02 ± 7.53	27.55 ± 4.85
8	763	MGDG 16:0/17:2	[M+Na]^+^	62.47 ± 25.38	62.50 ± 12.49	74.34 ± 21.78	50.41 ± 9.38 *
9	773	MGDG 16:1/18:3	[M+Na]^+^	138.53 ± 27.91	144.13 ± 23.07	156.02 ± 16.21	155.10 ± 16.57
10	775	MGDG 16:0/18:3	[M+Na]^+^	398.62 ± 56.63	325.94 ± 64.11	403.47 ± 70.95	311.94 ± 37.64 *
11	777	MGDG 16:0/18:2	[M+Na]^+^	124.28 ± 17.37	93.59 ± 8.26 *	128.74 ± 18.99	124.63 ± 25.76
12	779	MGDG 16:0/18:1	[M+Na]^+^	2.51 ± 1.98	28.26 ± 9.28 *	3.29 ± 2.87	17.50 ± 3.62 *
13	799	MGDG 18:2/18:3	[M+Na]^+^	68.62 ± 4.81	84.78 ± 9.63 *	89.77 ± 28.35	77.90 ± 22.35
14	801	MGDG 18:2/18:2	[M+Na]^+^	471.10 ± 45.19	586.25 ± 93.75 *	593.17 ± 226.25	551.62 ± 177.46
15	803	MGDG 18:1/18:2	[M+Na]^+^	697.14 ± 50.39	885.31 ± 93.27 *	923.49 ± 276.11	802.00 ± 177.73
16	805	MGDG 16:0/20:2	[M+Na]^+^	123.93 ± 9.75	166.84 ± 20.60 *	154.72 ± 53.53	136.05 ± 30.75
17	807	MGDG 16:0/20:1	[M+Na]^+^	4.34 ± 2.39	6.72 ± 1.67 *	9.17 ± 4.47	4.67 ± 1.07 *
18	809	MGDG 16:0/20:0	[M+Na]^+^	87.78 ± 64.26	77.33 ± 49.96	171.74 ± 88.79	114.71 ± 27.39
19	823	MGDG 16:0/21:0	[M+Na]^+^	67.66 ± 9.15	68.43 ± 21.47	110.69 ± 50.04	109.86 ± 44.94
	Phytyl Derivatives						
20	871	Pheophytin a	[M+H]^+^	522.39 ± 106.64	631.85 ± 435.75	75.07 ± 15.69	127.23 ± 29.06 *
21	893	Chlorophyll a	[M+H]^+^	157.53 ± 18.27	170.56 ± 58.57	111.87 ± 27.99	195.90 ± 35.34 *
	Negative ion mode						
	Phosphatidylglycerol (PG)						
22	721	PG 16:0/16:0	[M-H]^−^	227.74 ± 40.19	293.08 ± 19.32 *	241.82 ± 40.15	286.54 ± 12.40 *
23	731	PG 16:0/17:2	[M-H]^−^	25.42 ± 3.40	46.78 ± 3.57 *	30.48 ± 4.38	16.14 ± 2.14 *
24	733	PG 16:0/17:1	[M-H]^−^	28.81 ± 4.06	24.00 ± 1.76 *	32.20 ± 5.52	26.12 ± 6.61
25	743	PG 16:0/18:3	[M-H]^−^	184.59 ± 40.43	79.70 ± 18.21 *	52.14 ± 20.42	40.78 ± 17.32
26	745	PG 16:0/18:2	[M-H]^−^	1790.41 ± 429.45	1186.95 ± 268.70 *	718.15 ± 280.60	560.95 ± 240.69
27	747	PG 16:0/18:1	[M-H]^−^	554.97 ± 96.70	658.61 ± 144.52	242.65 ± 93.41	283.00 ± 117.93
28	749	PG 16:0/18:0	[M-H]^−^	46.74 ± 7.80	16.78 ± 4.92 *	15.53 ± 7.27	25.90 ± 8.04 *
	Sulfoquinvosyldiacylglycerol (SQDG)						
29	763	SQDG 14:1/16:0	[M-H]^−^	20.00 ± 4.76	6.12 ± 6.63 *	10.46 ± 4.59	11.58 ± 4.62
30	765	SQDG 14:0/16:0	[M-H]^−^	102.57 ± 24.01	21.46 ± 10.69 *	62.01 ± 22.80	34.26 ± 11.14 *
31	747	SQDG 14:0/18:3	[M-H]^−^	37.78 ± 5.94	18.11 ± 3.12 *	28.44 ± 6.26	20.92 ± 8.70
32	789	SQDG 14:0/18:2	[M-H]^−^	88.71 ± 20.79	34.51 ± 5.15 *	42.64 ± 17.24	28.61 ± 14.09
33	791	SQDG 16:0/16:1	[M-H]^−^	1344.99 ± 318.03	878.52 ± 198.49 *	738.59 ± 286.80	571.89 ± 227.26
34	793	SQDG 16:0/16:0	[M-H]^−^	3495.78 ± 770.70	1810.00 ± 480.7 *	1790.48 ± 691.01	1184.24 ± 469.66 *
35	803	SQDG 16:0/17:2	[M-H]^−^	95.83 ± 23.92	27.00 ± 6.81 *	34.67 ± 13.94	16.27 ± 6.85 *
36	805	SQDG 16:0/17:1	[M-H]^−^	311.37 ± 50.70	246.53 ± 63.70	160.00 ± 66.82	123.67 ± 50.69
37	807	SQDG 16:0/17:0	[M-H]^−^	198.33 ± 42.89	112.62 ± 32.15 *	79.49 ± 31.17	44.82 ± 18.63 *
38	813	SQDG 16:0/18:4	[M-H]^−^	377.56 ± 64.81	265.30 ± 9.52 *	288.50 ± 38.10	258.27 ± 22.99
39	815	SQDG 16:0/18:3	[M-H]^−^	1020.36 ± 267.61	359.39 ± 56.36 *	582.38 ± 204.28	501.31 ± 179.22
40	817	SQDG 16:0/18:2	[M-H]^−^	6748.15 ± 1825.81	3287.51 ± 883.17 *	3039.48 ± 1152.15	2330.66 ± 1022.07
41	819	SQDG 16:0/18:1	[M-H]^−^	4661.42 ± 986.36	7023.10 ± 1866.88 *	3189.82 ± 1209.63	3086.93 ± 1167.90
42	821	SQDG 16:0/18:0	[M-H]^−^	1056.37 ± 253.67	1495.41 ± 398.34 *	742.22 ± 275.01	655.08 ± 243.36
43	831	SQDG 17:0/18:2	[M-H]^−^	38.98 ± 7.94	14.79 ± 4.11 *	15.61 ± 6.32	11.23 ± 3.93
44	835	SQDG 16:0/19:0	[M-H]^−^	64.58 ± 23.80	38.91 ± 11.20 *	26.79 ± 8.83	31.14 ± 16.67
45	839	SQDG 18:2/18:3	[M-H]^−^	15.14 ± 4.08	5.79 ± 0.77 *	9.84 ± 2.04	8.70 ± 1.23
46	841	SQDG 18:1/18:3	[M-H]^−^	24.30 ± 5.66	9.29 ± 3.13 *	15.71 ± 5.42	11.56 ± 4.08
47	843	SQDG 16:0/20:3	[M-H]^−^	76.68 ± 20.50	36.40 ± 8.59 *	48.27 ± 16.34	46.82 ± 17.52
48	845	SQDG 18:0/18:2	[M-H]^−^	83.52 ± 21.38	47.71 ± 12.15 *	48.03 ± 16.39	48.75 ± 19.35
49	847	SQDG 18:0/18:1	[M-H]^−^	49.56 ± 14.03	58.07 ± 15.49 *	23.99 ± 8.64	33.75 ± 11.66
50	849	SQDG 16:0/20:0	[M-H]^−^	28.34 ± 8.74	39.63 ± 9.09	10.23 ± 3.41	16.25 ± 5.92 *

^1^ Values are represented as means ± standard deviations (*n* = 9, biological triplicates and experimental triplicates) after normalization with IS. ^2^ Asterisks (*) represent significant differences compared with each control on days 9 and 18, *p* < 0.05 (from Mann–Whitney test).

**Table 4:** Relative yields (relative intensity/L) of selected metabolites and intact lipid species from PLS-DA-derived biplots with samples from the control and 5-mM exogenous glucose treatment groups.

No.	Compounds	Day 9		No.	Compounds	Day 18	
		Control	5-mM Glucose			Control	5-mM Glucose
1	Aspartic acid	11.25 ± 2.33	50.99 ± 8.03 *^,1^	1	Chlorophyll a	126.91 ± 31.78	146.36 ± 25.32
2	Fructose	ND ^5^	0.35 ± 0.07	2	DGDG ^3^ 16:0/18:1	15.99 ± 2.25	16.72 ± 2.07
3	Glucosamine	ND	0.34 ± 0.02	3	Glutamic acid	58.27 ± 6.36	70.65 ± 10.14
4	Glutamic acid	18.48 ± 6.24	93.63 ± 6.38 *	4	MGDG 16:0/18:1	5.45 ± 4.81	12.45 ± 2.25 *
5	Glycerol-3-phosphate	10.99 ± 2.61	31.78 ± 4.52 *	5	Neophytadiene	5.19 ± 0.73	6.72 ± 0.58 *
6	Linolenic acid	0.34 ± 0.10	1.29 ± 0.18 *	6	Oleic acid	5.48 ± 1.42	6.49 ± 0.94
7	MGDG ^2^ 16:0/18:1	1.00 ± 0.76	22.94 ± 7.35 *	7	Pheophytin a	85.38 ± 19.61	95.60 ± 22.08
8	MGDG 16:0/20:2	50.84 ± 3.35	135.95 ± 18.25 *	8	Pyroglutamic acid	7.84 ± 1.26	8.08 ± 1.00
9	MGDG 18:1/18:2	286.87 ± 29.23	721.19 ± 82.32 *	9	Succinic acid	0.68 ± 0.45	1.65 ± 0.36 *
10	Neophytadiene	2.14 ± 0.33	8.94 ± 0.87 *	10	Sucrose	200.79 ± 21.54	216.88 ± 24.23
11	Oleic acid	0.83 ± 0.18	7.69 ± 1.53 *				
12	PG ^4^ 16:0/16:0	93.85 ± 19.02	238.55 ± 15.61 *				
13	PG 16:0/17:2	10.42 ± 1.25	38.06 ± 2.75 *				
14	Pyroglutamic acid	2.38 ± 0.29	5.69 ± 0.81 *				
15	Sucrose	25.31 ± 11.75	96.05 ± 19.30 *				

^1^ The asterisks (*) indicate significant differences compared with each control on days 9 and 18, *p* < 0.05 (from Mann–Whitney test). ^2^ MGDG, monogalactosyldiacylglycerol. ^3^ DGDG, digalactosyldiacylglycerol. ^4^ PG, phosphatidylglycerol, ^5^ ND, not detected.

**Table 5:** Relative yields (relative intensity/L) of metabolites and intact lipid species identified equally from PLS-DA-derived biplots with samples from days 9 and 18 of the 5-mM glucose group.

No.	Compounds	5-mM Glucose	
		Day 9	Day 18
1	Glutamic acid	93.63 ± 6.38	70.65 ± 10.14 *^,1^
2	MGDG ^2^ 16:0/18:1	22.94 ± 7.35	12.45 ± 2.25 *
3	Neophytadiene	8.94 ± 0.87	6.72 ± 0.58 *
4	Oleic acid	7.69 ± 1.53	6.49 ± 0.94 *
5	Pyroglutamic acid	5.69 ± 0.81	8.08 ± 1.00 *
6	Sucrose	96.05 ± 19.30	216.88 ± 24.23 *

^1^ The asterisk (*) indicates significant differences in relative yields in the 5-mM glucose group between days 9 and 18, *p* < 0.05 (from Mann–Whitney test). ^2^ MGDG, monogalactosyldiacylglycerol.

## Data Availability

The data presented in this study are available on request from the corresponding author.

## Supplementary Materials

The following are available online at https://www.mdpi.com/2218-273X/11/2/214/s1, Figure S1: MS/MS spectra of the identified nine intact lipid species (A-I) in *Synechocystis* sp. PCC 7338 matched to libraries, Figure S2: PCA-derived score plot of *Synechocystis* 7338 in the control and 5-mM exogenous glucose treatments with quality control (QC), Figure S3: PLS-DA-derived score plots with samples from days 9 (A) and 18 (B) and diagram of permutation test from days 9 (C) and 18 (D) of *Synechocystis* 7338 in the control and 5-mM exogenous glucose treatments, Table S1: List of metabolites identified in *Synechocystis* sp. PCC 7338 by GC-MS analysis.
