# Supplementary material for: Enhanced Production of Photosynthetic Pigments and Various Metabolites and Lipids in the Cyanobacteria Synechocystis sp. PCC 7338 Culture in the Presence of Exogenous Glucose
Source: Biomolecules. 2021 Feb 3;11(2):214. doi: 10.3390/biom11020214 (PMC7913732; doi:10.3390/biom11020214)
Supplement: Supplementary file 1 [file biomolecules-11-00214-s001.pdf]

*Supplementary data*

# Enhanced Production of Photosynthetic Pigments and Various Metabolites and Lipids in the Cyanobacteria *Synechocystis* sp. PCC 7338 Culture in the Presence of Exogenous Glucose

**YuJin Noh [1,†], Hwanhui Lee[1,†], Myeongsun Kim[1], Seong-Joo Hong[2], Hookeun Lee[3], Dong-Myung Kim[4], Byung-Kwan Cho[5], Choul-Gyun Lee[2], Hyung-Kyoon Choi[1,*]**

[1] College of Pharmacy, Chung-Ang University, Seoul 06974, Republic of Korea; n5uuu@naver.com (Y.J.N.); hwanhui56@gmail.com (H.L.); myeongsunkim0242@gmail.com (M.K.)
[2] Department of Biological Engineering, Inha University, Incheon 22212, Republic of Korea; owlet77@gmail.com (S.J.H); leecg@inha.ac.kr (C.G.L)
[3] College of Pharmacy, Gachon University, Incheon 13120, Republic of Korea; hklee@gachon.ac.kr (H.L.)
[4] Department of Chemical Engineering and Applied Chemistry, Chungnam National University, Daejeon 34134, Republic of Korea; dmkim@cnu.ac.kr (D.M.K.)
[5] Department of Biological Sciences, KAIST, Daejeon 34141, Republic of Korea; bcho@kaist.ac.kr (B.K.C.)
* Correspondence: hykychoi@cau.ac.kr; Tel.: +82-2-820-5605
† These authors equally contribute to this work.

**Citation:** Noh, Y.; Lee, H.; Kim, M.; Hong, S.-J.; Lee, H.; Kim, D.-M.; Cho, B.-K.; Lee, C.-G.; Choi, H.-K. Enhanced production of photosynthetic pigments and various metabolites and lipids in the cyanobacteria *Synechocystis* sp. PCC 7338 culture in the presence of exogenous glucose. **2021**, *10*, x. https://10.3390/biom11020214

Received: 17 December 2020
Accepted: 29 January 2021
Published: 03 February 2021

**Publisher's Note:** MDPI stays neutral with regard to jurisdictional claims in published maps and institutional affiliations.

**Copyright:** © 2021 by the authors. Submitted for open access publication under the terms and conditions of the Creative Commons Attribution (CC BY) license (http://creativecommons.org/licenses/by/4.0/).

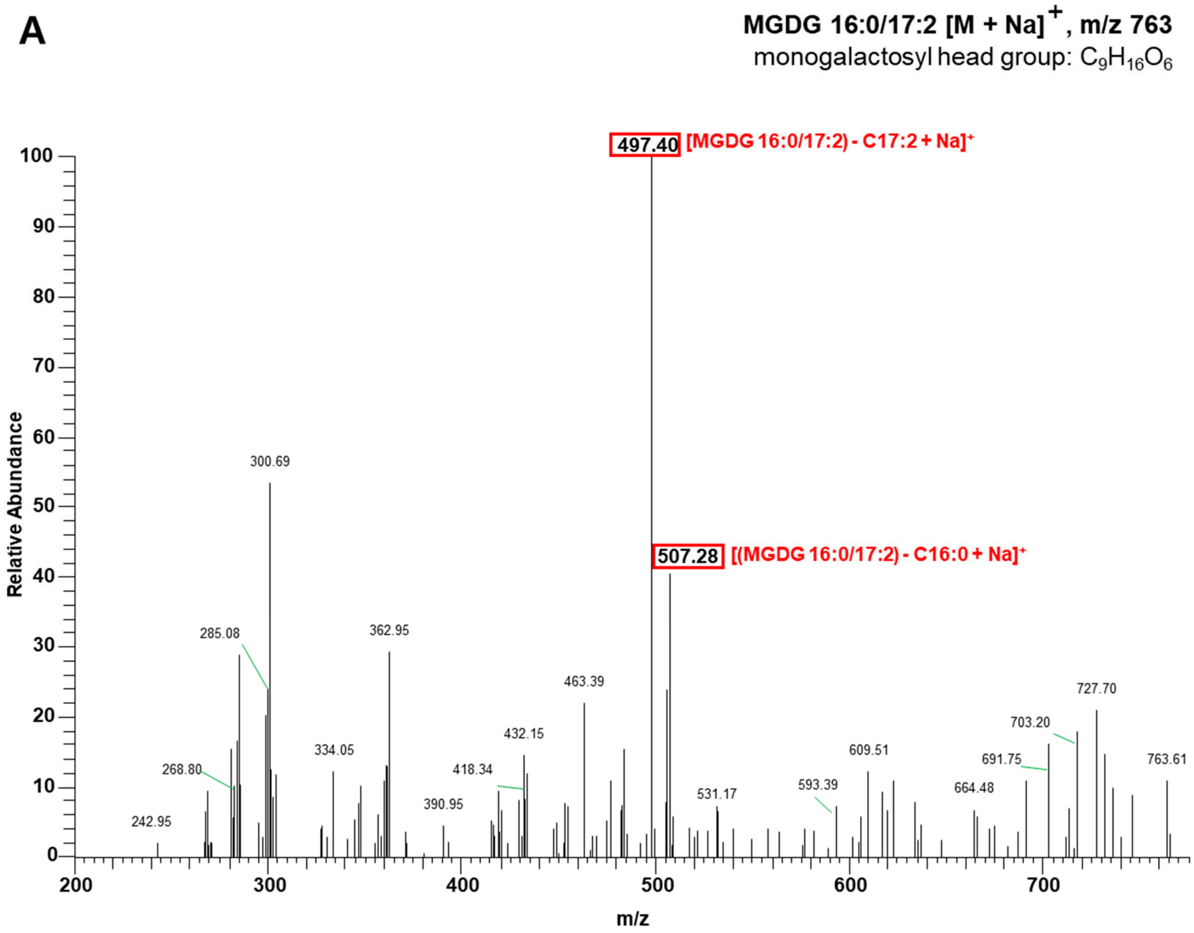


**Figure S1.** The MS/MS spectra of the identified nine intact lipid species (**A**-**I**) in *Synechocystis* sp. PCC 7338 matched to libraries (the authentic references, data base of LIPID MAPS, LipidBlast, and an in-house MS/MS library). The major precursor and fragment ions were indicated red characters. MGDG, monogalactosyldiacylglycerol; DGDG, digalactosyldiacylglycerol; PG, phosphatidylglycerol; SQDG, sulfoquinovosyldiacylglycerol.

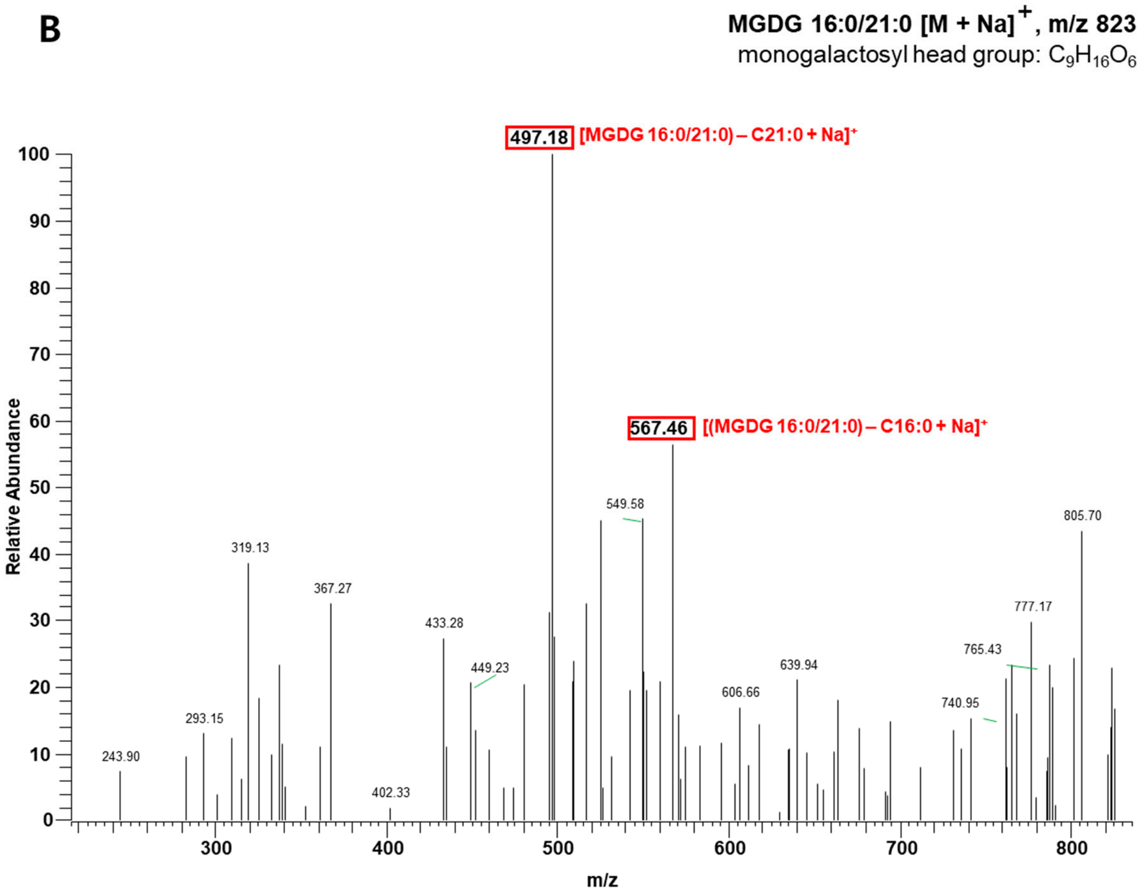
B
MGDG 16:0/21:0 [M + Na]+, m/z 823
monogalactosyl head group: C9H16O6
497.18 [MGDG 16:0/21:0) – C21:0 + Na]+
567.46 [(MGDG 16:0/21:0) – C16:0 + Na]+
549.58
805.70
319.13
367.27
433.28
449.23
777.17
765.43
639.94
606.66
740.95
293.15
243.90
402.33
Relative Abundance
m/z

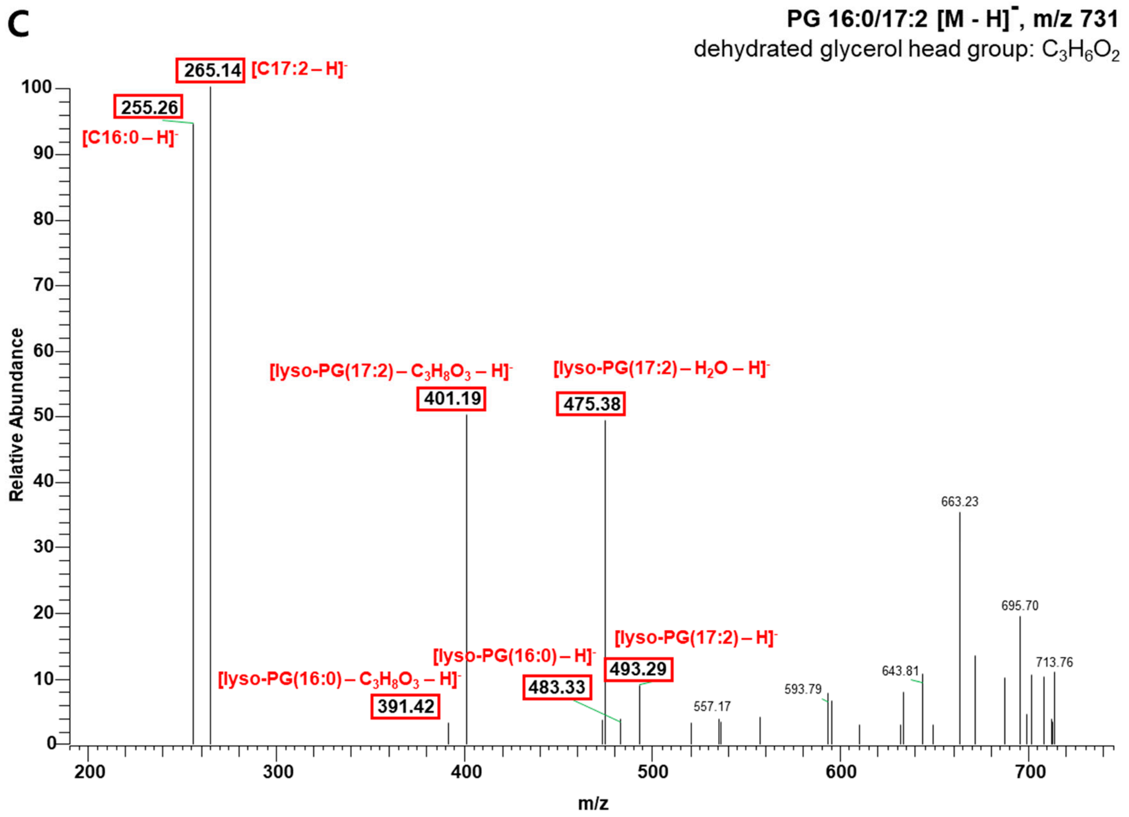
C
PG 16:0/17:2 [M - H]-, m/z 731
dehydrated glycerol head group: C3H6O2
265.14 [C17:2 – H]-
255.26
[C16:0 – H]-
[lyso-PG(17:2) – C3H8O3 – H]-
401.19
[lyso-PG(17:2) – H2O – H]-
475.38
663.23
695.70
713.76
[lyso-PG(17:2) – H]-
[lyso-PG(16:0) – H]-
493.29
483.33
[lyso-PG(16:0) – C3H8O3 – H]-
391.42
557.17
593.79
643.81
Relative Abundance
m/z

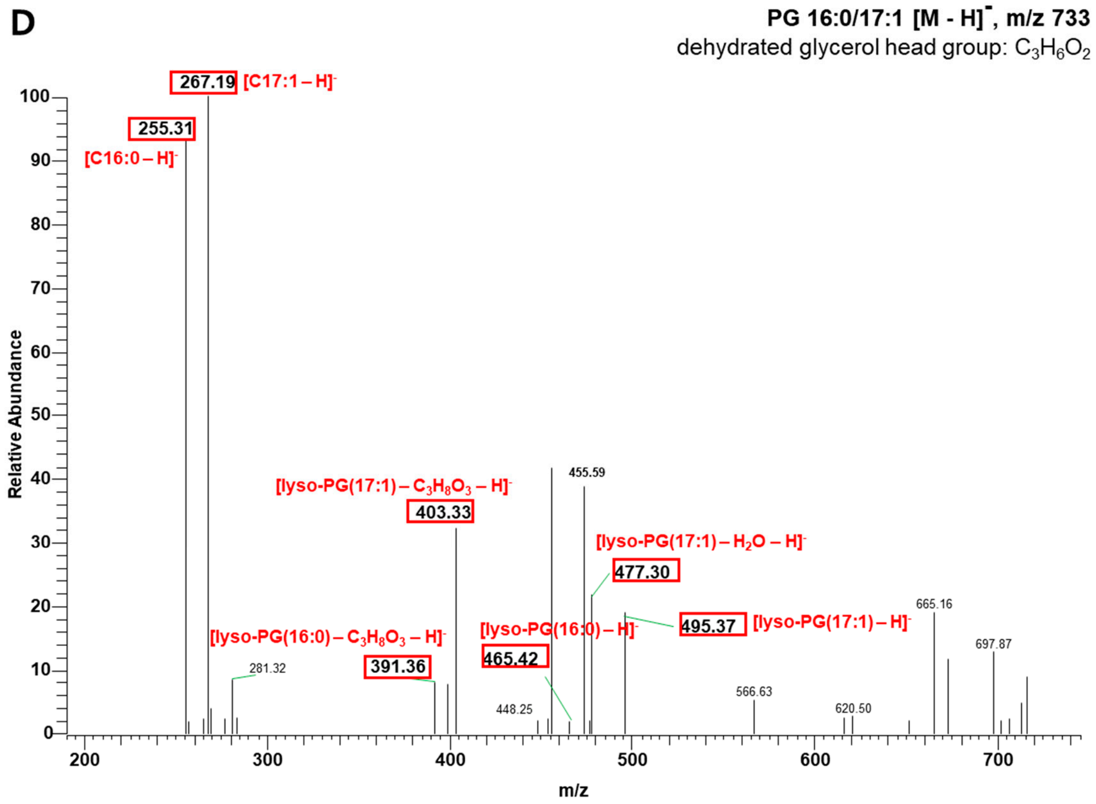
D
PG 16:0/17:1 [M - H]-, m/z 733
dehydrated glycerol head group: C3H6O2
267.19 [C17:1 – H]-
255.31
[C16:0 – H]-
[lyso-PG(17:1) – C3H8O3 – H]-
403.33
455.59
[lyso-PG(17:1) – H2O – H]-
477.30
665.16
[lyso-PG(16:0) – H]-
495.37 [lyso-PG(17:1) – H]-
[lyso-PG(16:0) – C3H8O3 – H]-
697.87
465.42
281.32
391.36
448.25
566.63
620.50
Relative Abundance
m/z

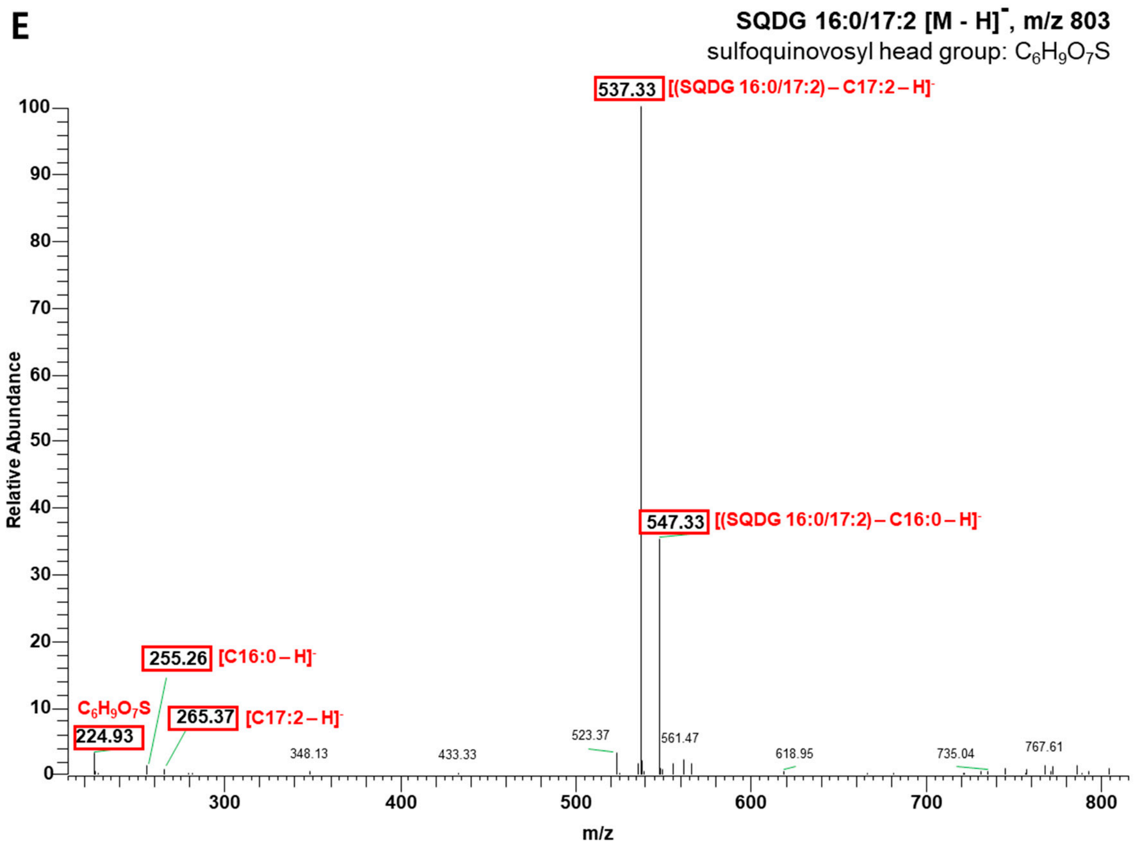
E
SQDG 16:0/17:2 [M - H]-, m/z 803
sulfoquinovosyl head group: C6H9O7S
537.33 [(SQDG 16:0/17:2) – C17:2 – H]-
547.33 [(SQDG 16:0/17:2) – C16:0 – H]-
255.26 [C16:0 – H]-
265.37 [C17:2 – H]-
C6H9O7S
224.93
348.13
433.33
523.37
561.47
618.95
735.04
767.61
Relative Abundance
m/z
100
90
80
70
60
50
40
30
20
10
0
300
400
500
600
700
800

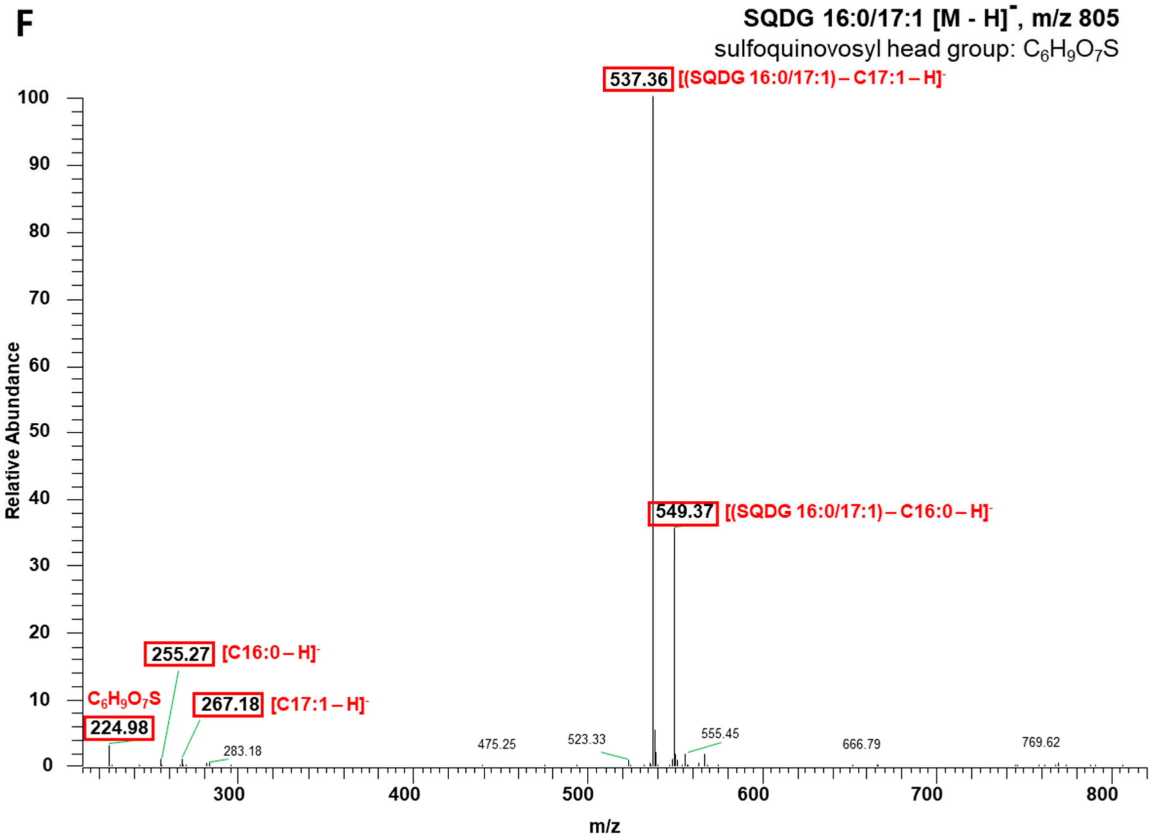
F
SQDG 16:0/17:1 [M - H]-, m/z 805
sulfoquinovosyl head group: C6H9O7S
537.36 [(SQDG 16:0/17:1) – C17:1 – H]-
549.37 [(SQDG 16:0/17:1) – C16:0 – H]-
255.27 [C16:0 – H]-
C6H9O7S
224.98
267.18 [C17:1 – H]-
283.18
475.25
523.33
555.45
666.79
769.62
Relative Abundance
m/z

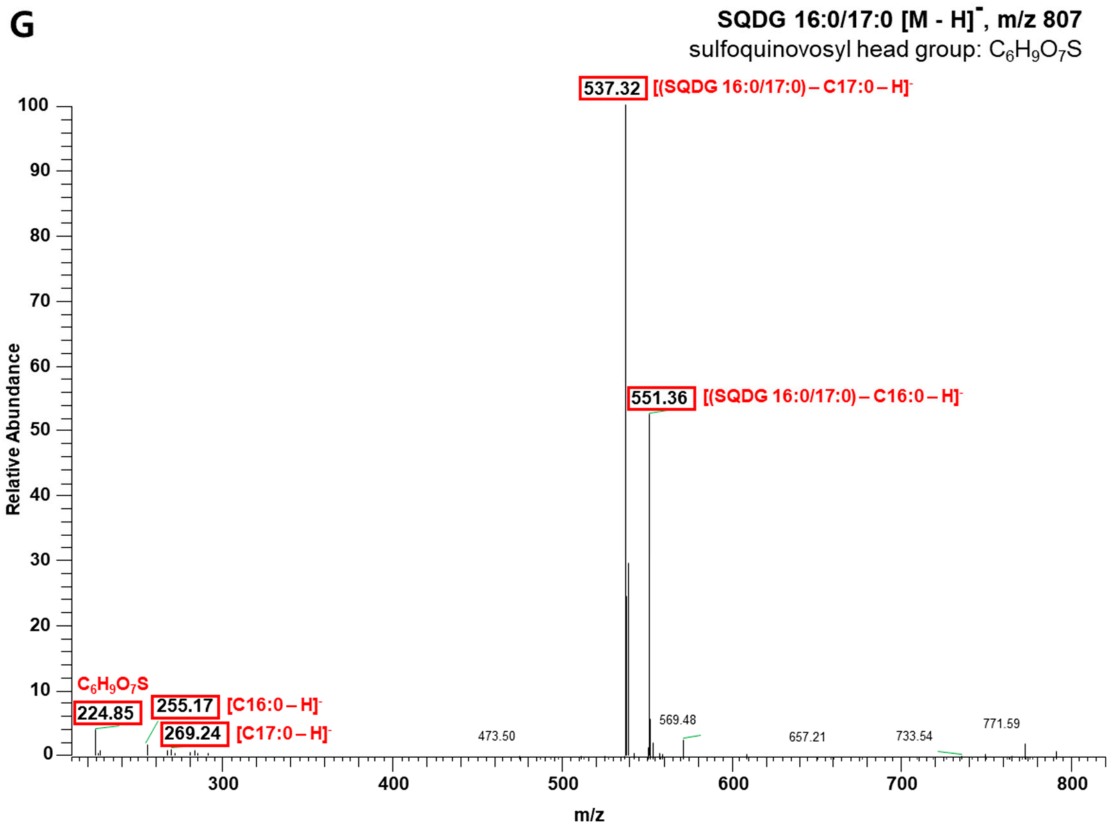
G
SQDG 16:0/17:0 [M - H]⁻, m/z 807
sulfoquinovosyl head group: C6H9O7S
537.32 [(SQDG 16:0/17:0) – C17:0 – H]⁻
551.36 [(SQDG 16:0/17:0) – C16:0 – H]⁻
C6H9O7S
224.85
255.17 [C16:0 – H]⁻
269.24 [C17:0 – H]⁻
473.50
569.48
657.21
733.54
771.59
Relative Abundance
m/z

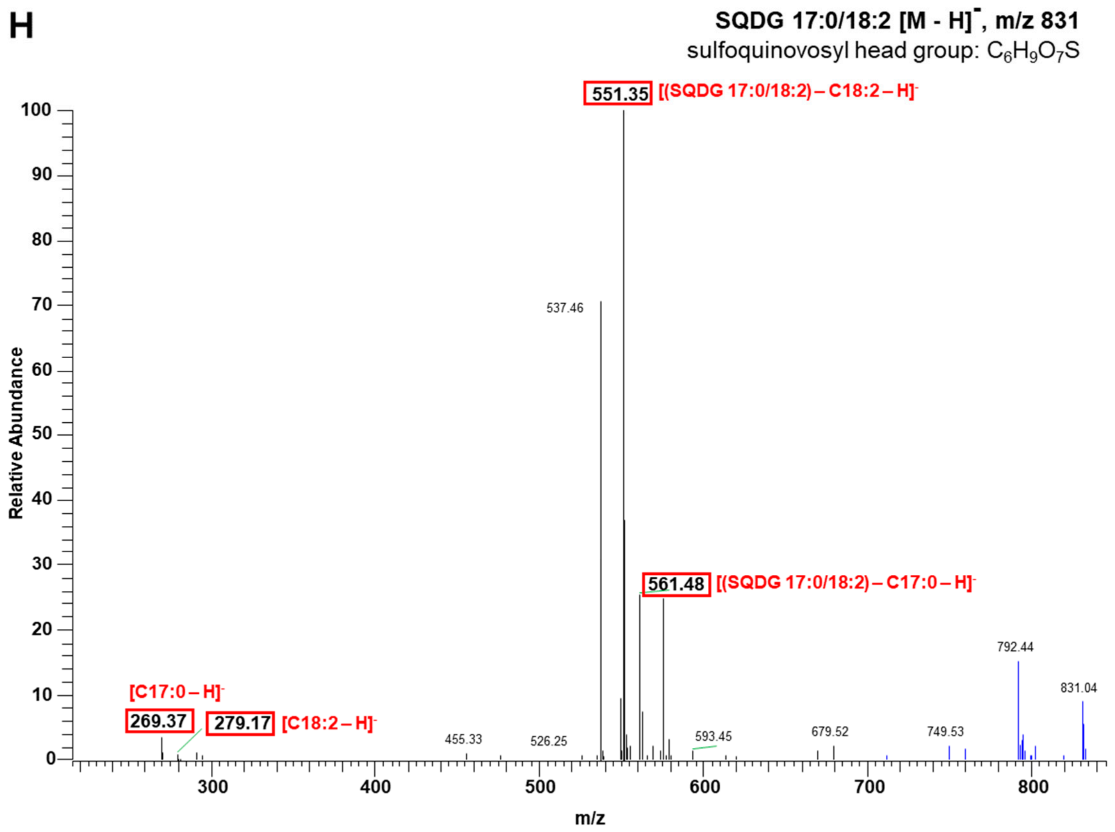
H
SQDG 17:0/18:2 [M - H]-, m/z 831
sulfoquinovosyl head group: C6H9O7S
551.35 [(SQDG 17:0/18:2) – C18:2 – H]-
537.46
561.48 [(SQDG 17:0/18:2) – C17:0 – H]-
792.44
831.04
[C17:0 – H]-
269.37
279.17 [C18:2 – H]-
455.33
526.25
593.45
679.52
749.53
Relative Abundance
m/z

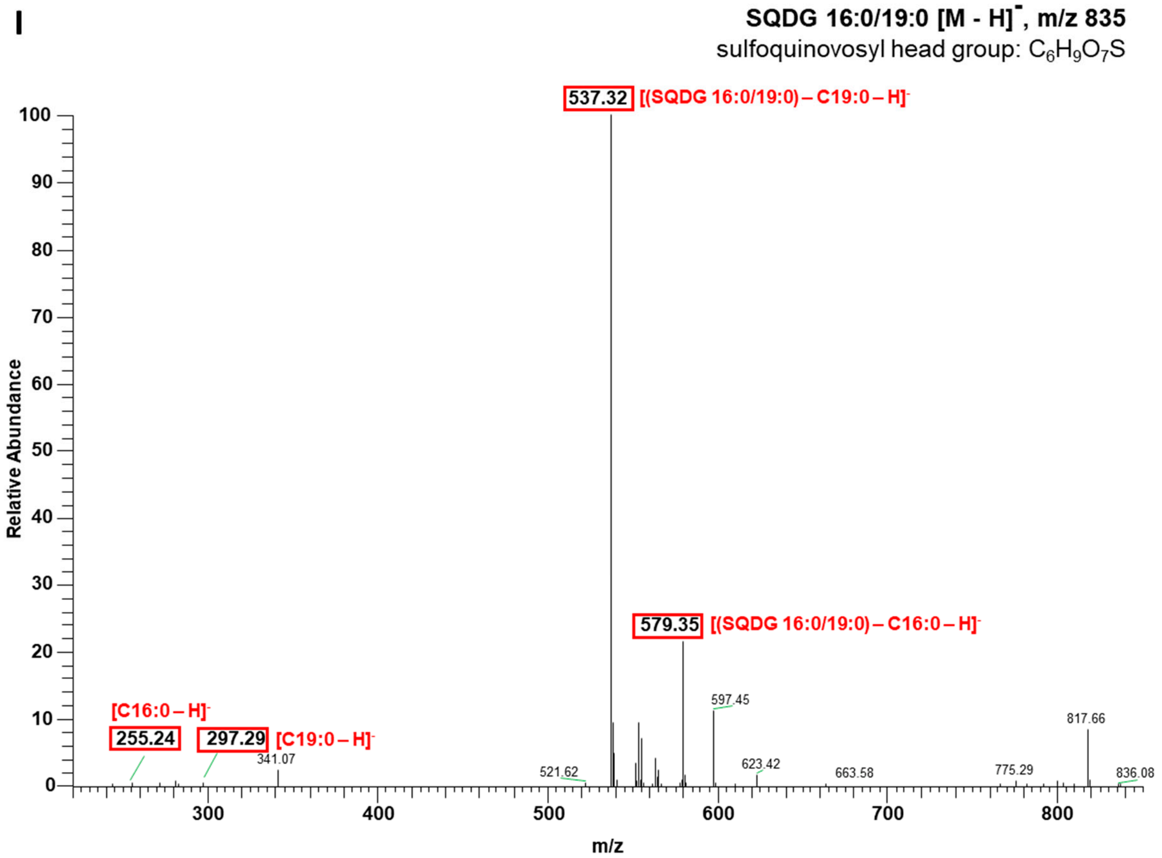
I
SQDG 16:0/19:0 [M - H]⁻, m/z 835
sulfoquinovosyl head group: C6H9O7S
537.32 [(SQDG 16:0/19:0) – C19:0 – H]-
579.35 [(SQDG 16:0/19:0) – C16:0 – H]-
[C16:0 – H]-
255.24
297.29 [C19:0 – H]-
341.07
521.62
597.45
623.42
663.58
775.29
817.66
836.08
Relative Abundance
m/z

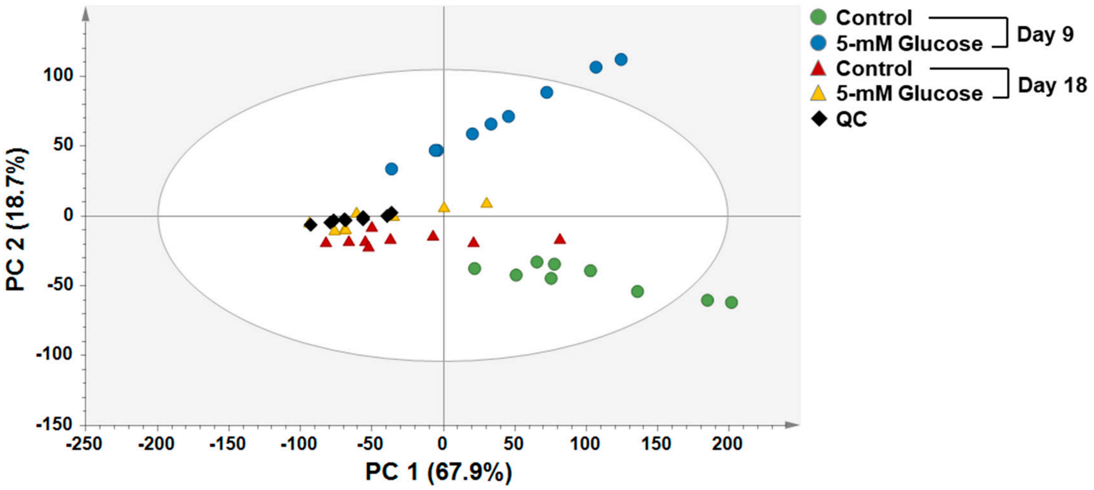


**Figure S2.** PCA-derived score plot of *Synechocystis* 7338 in the control and 5-mM exogenous glucose treatments with quality control (QC).

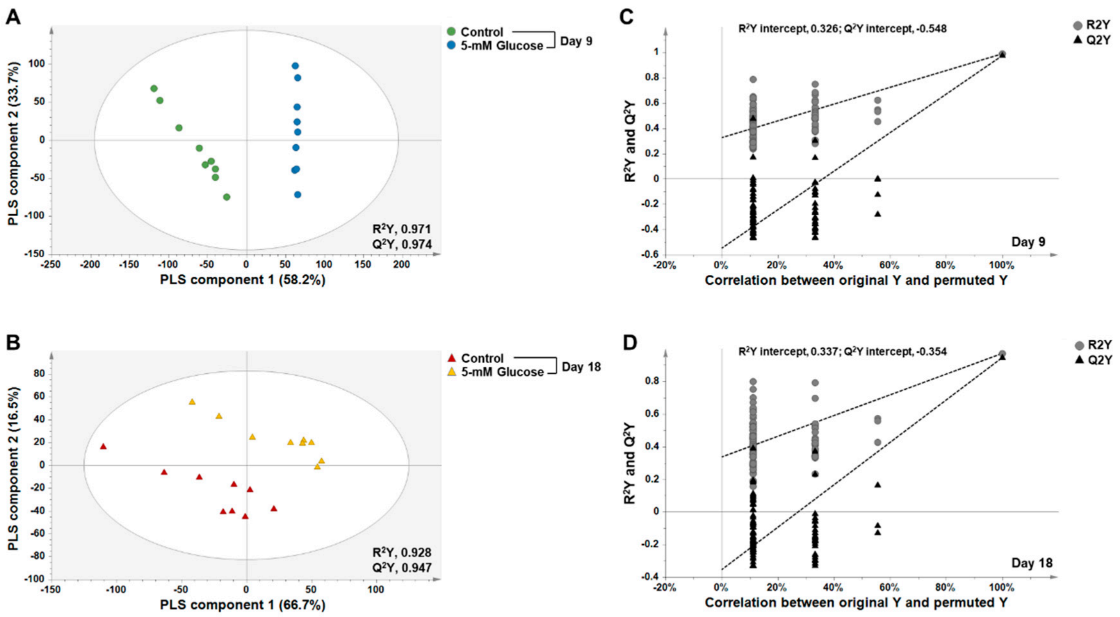


**Figure S3.** PLS-DA-derived score plots with samples from days 9 (**A**) and 18 (**B**) and diagram of permutation test from days 9 (**C**) and 18 (**D**) of *Synechocystis* 7338 in the control and 5-mM exogenous glucose treatments (*n* = 9, biological triplicates and experimental triplicates).

**Table S1.** List of metabolites identified in *Synechocystis* sp. PCC 7338 by GC-MS analysis.

| No. | Compound | m/z | RT | Mass fragment (m/z) | TMS |
|---|---|---|---|---|---|
| | **Alcohols** | | | | |
| 1 | Glycerol | 205 | 13.36 | 103, 117, 133, **205** | 3 |
| 2 | Glycerol-3-phosphate | 357 | 25.1 | 299, 315, **357**, 445 | 4 |
| | **Amino acids** | | | | |
| 3 | Alanine | 116 | 8.79 | 100, **116**, 190, 218 | 2 |
| 4 | Aspartic acid | 160 | 17.25 | 116, 117, 130, **160** | 2 |
| | | 232 | 19.62 | 100, 202, 218, **232** | 3 |
| | | | 19.64 | | |
| 5 | Glutamic acid | 246 | 21.99 | 128, 156, **246**, 348 | 3 |
| 6 | Glycine | 174 | 14.14 | 86, **174**, 248, 276 | 3 |
| 7 | Pyroglutamic acid | 156 | 19.56 | 133, **156**, 230, 258 | 2 |
| 8 | Serine | 132 | 12.87 | 103, 113, **132**, 159 | 2 |
| | **Fatty acids** | | | | |
| 9 | Linoleic acid | 337 | 33.76 | 81, 95, 129, **337** | 1 |
| 10 | Linolenic acid | 79 | 33.41 | **79**, 91, 129, 335 | 1 |
| 11 | Oleic acid | 339 | 33.89 | 117, 129, 145, **339** | 1 |
| 12 | Palmitic acid | 313 | 30.82 | 117, 129, 132, **313** | 1 |
| 13 | Palmitoleic acid | 311 | 30.41 | 117, 129, 145, **311** | 1 |
| 14 | Stearic acid | 341 | 34.36 | 117, 129, 132, **341** | 1 |
| | **Glycerolipids** | | | | |
| 15 | 1-Monopalmitin | 371 | 39.87 | 129, 205, **371**, 459 | 2 |
| 16 | Glycerol monosterate | 399 | 42.68 | 129, 205, **399**, 487 | 2 |
| | **Organic acids** | | | | |
| 17 | Isocitric acid | 273 | 26.37 | 245, **273**, 319, 465 | 4 |
| 18 | Lactic acid | 117 | 7.69 | **117**, 133, 191, 219 | 2 |
| 19 | Succinic acid | 247 | 14.43 | 129, 133, 172, **247** | 2 |
| | **Sugars** | | | | |
| 20 | Fructose | 217 | 26.16 | 103, 204, **217**, 437 | 5 |
| | | 103 | 27.55 | **103**, 217, 277, 307 | 6 (MeOX) |
| | | | 27.35 | | |
| 21 | Glucosamine | 203 | 29 | 131, **203**, 217, 304 | 6 |
| 22 | Glucose | 204 | 27.76 | 129, 191, **204**, 217 | 5 |
| | | | 29.52 | | |
| 23 | Glucose-6-phosphate | 387 | 36.51 | 204, 299, 357, **387** | 6 |
| | | | 36.7 | | |
| 24 | Glucosylglycerol | 204 | 34.62 | 103, **204**, 337, 361 | 6 |
| | | | 36.19 | | |
| 25 | Sucrose | 361 | 40.52 | 103, 217, **361**, 437 | 8 |
| | **Others** | | | | |
| 26 | Neophytadiene | 68 | 26.73 | **68**, 82, 95, 123 | 0 |
| 27 | Heptadecane | 57 | 23.84 | **57**, 71, 85, 99 | 0 |

RT, retention time; TMS, trimethylsylation; MeOX, methoxyamination; Bold character in mass fragment, the most intensive peak in a mass spectrum.
